# Climate Change and Emotions: Analysis of People’s Emotional States in Southern Ecuador

**DOI:** 10.3389/fpsyg.2021.644240

**Published:** 2021-09-27

**Authors:** Verónica Iniguez-Gallardo, Daniela Lenti Boero, Joseph Tzanopoulos

**Affiliations:** ^1^Manejo y Gestión de Recursos Naturales, Departamento de Ciencias Biológicas, Universidad Técnica Particular de Loja, Loja, Ecuador; ^2^Durrell Institute of Conservation and Ecology, Kent Interdisciplinary Centre for Spatial Studies, University of Kent, Canterbury, United Kingdom; ^3^Independent Researcher, Valle d’Aosta, Italy

**Keywords:** climate change, emotions, Ecuador, open-ended questions, qualitative and quantitative analysis

## Abstract

Climate change involves multiple emotional expressions associated with specific labels, notably: ‘concern,’ ‘guilt,’ or ‘scepticism.’ However, there are other types of emotions that have been less analysed, such as ‘powerlessness,’ ‘anger’ and ‘confusion’ that are of equal importance for predicting behavioural changes toward this climatic issue. Likewise, few studies in this research field rely on qualitative data to understand and identify the causative agents for the emotional arousal. This research explores a range of emotions, mixing those that have been widely studied and those that have been hardly analysed. It also looks at the demographic parameters associated with such emotions using a population sample from southern Ecuador. The study analyses quantitative and qualitative data gathered through structured-questionnaires whereby participants were given agency to select and define how they themselves sense emotionally climate change. The results indicate that two of the five participants’ most selected emotions are shared with other nations (‘concern,’ ‘guilt’), while the other three have been less reported and studied in the climate change field (‘powerlessness,’ ‘anger,’ and ‘confusion’). These emotions were found to be aroused by different reasons associated with specific demographic variables. The findings reveal the role of the cultural and local environment in the emotional arousal and its relevance for designing more effective climate communication campaigns.

## Introduction

Climate change has generated a considerable social debate involving cultural practices and context, social beliefs, cognitive representations, and emotional reactions that need to be studied in order to adopt individual and collective practices that reduce CO_2_ emissions and enhance adaptation to new climatic conditions. Of these aspects, emotions are the starting point of human actions in that they influence thinking and learning at the individual or collective level (Moser, 2010; Caillaud et al., 2016), as well as drive people to anticipate or avoid actions that lead to anti-social behaviour (Baumeister and Bushman, 2011). Emotions are also decisive drivers in the cognitive decision-making process (Lerner et al., 2015) and action performance (Lewis, 2005). In the climate context it is claimed that, beyond perception, understanding emotional reactions is pivotal to enhance human practices and actions that are relevant to adaptation and mitigation responses to climate change (Corral-Verdugo, 2021) in that unattended feelings can be maladaptive and misleading, e.g., a hopeless fear may drive to numbness and inaction instead of promoting good practices (Moser, 2010; Roeser, 2012). The analysis of emotional reactions induced by the cognitive appraisal of climate change is, therefore, relevant for predicting the behavioural changes required in addressing climate change.

This research field is relatively recent and has been growing steadily over the last decade with most of the studies being conducted in Europe and the United States. The contributions of these studies are remarkable and have made it possible to identify a range of emotions experienced by the public in relation to the current climate crisis. The most commonly emotional states that have been identified are ‘alarm,’ ‘concern,’ ‘doubt,’ ‘dismissive,’ ‘scepticism’ (Brulle et al., 2012; Leombruni, 2015; Shi et al., 2016; Leiserowitz et al., 2020), ‘anger,’ ‘sadness,’ ‘guilt’ (Smith and Leiserowitz, 2014; Chu and Yang, 2019), ‘hope,’ ‘fear,’ ‘anxiety,’ ‘compassion,’ ‘worry’ (Myers et al., 2012; Ojala, 2015; Stevenson and Peterson, 2016; Nabi et al., 2018; Gustafson et al., 2020; Zummo et al., 2020) ‘anticipation,’ ‘disgust,’ and ‘surprise’ (Loureiro and Alló, 2020). Although identified in the climate context, ‘powerlessness,’ ‘confusion,’ and ‘happiness,’ have been less investigated (Aitken et al., 2011; Barnes et al., 2013; Loureiro and Alló, 2020), while other types of positive and passive emotions such as ‘optimism’ ‘calm’ and ‘indifference’ have not yet been reported nor analysed. Other types of studies draw conclusions from cross-national samples to analyse the impact of negative emotions on mental health (Ogunbode et al., 2021), or provide a profound analysis of the indirect psychological impacts of global warming by linking a considerable range of emotions to climate change such as ‘anxiety,’ ‘guilt,’ ‘despair,’ etc. (Doherty and Clayton, 2011). It is worth mentioning that these studies rely on structured questionnaires with close-ended questions designed to select a type of emotion or a phrase/message that refers to an emotion felt by the subjects, without collecting data on causal agents of the emotion. This latter aspect requires further research if we consider that in order to design climate campaigns and activities, it is of high relevance to obtain an in-depth understanding of the specific causal agents of the emotions felt (Moser, 2010).

Additionally, climate change as an emotional stimulus is completely different from those traditionally used in emotional research, both biological-neurological (Darwin, 1872; Ekman, 1992; James and Lange, 1922; Panksepp, 1998; LeDoux, 2000), and dimensional-constructivist/cognitive (e.g., Russell, 1980; Posner et al., 2005), where stimuli are in the sensorial and/or cognitive domain of the subjects and are directly experienced by them in experiments using, e.g., photographs, videos, music, odours, etc., or in their past life (research of situational antecedents of emotions, e.g., having a vacation). Climate change as a phenomenon (rising temperature is changing global climate), is investigated and fully understood only by specialists, while the appraisal of this phenomenon by the general public is related to a complex process, influenced by many factors such as:

(a) Belief in science: climate change is intangible and requires people to believe in scientific knowledge. Belief in science is actually reported as a consistent variable for different complex phenomena that only science explains such as climate change and Covid-19 (Brzezinski et al., 2020).

(b) Institutional intervention and/or media coverage: this helps making available to the general public scientific knowledge in a non-specialist and comprehensible way (Mercado, 2012; Boykoff, 2013; Jaspal and Nerlich, 2014; Graham and De Bell, 2020). Yet, it has been found that the media can manipulate grammatically or lexically climate messages causing uncertainty among the public about the ultimate causes of climate change (Bailey et al., 2014). Likewise, political orientation has also been associated with climate emotions (Brulle et al., 2012), with examples from Russia and Kazakhstan where government and media have played an important role in shaping a denial attitude about climate change (Graybill, 2012; Poberezhskaya, 2014; Poberezhskaya and Danilova, 2021).

(c) Understanding of epiphenomena of climate change: drought, hurricanes, hot summer, etc. are understood differently by the public. For example, farmers in developing countries such as India (Dhanya and Ramachandran, 2015; Singh, 2020), Thailand and Vietnam (Waibel et al., 2018), Ethiopia and South Africa (Bryan et al., 2009), and rural Sahel (Mertz et al., 2009), perceive climate-related impacts such as poor livestock health or reduce crop yields and adapt their farming activities accordingly. Such epiphenomena are not equal at all latitudes, and affect regions differently, and are therefore perceived and understood diversely (Iniguez-Gallardo et al., 2020). Likewise, the type of emotions that develop over these epiphenomena are determined by the appraisal or interpretation that people make about specific events or circumstances (Karasawa, 1995). Neglecting these understandings may lead to ineffective practices that might worsen the situation (Kemausuor et al., 2011).

The above suggests that climate emotions develop according to various demographic variables adjusted to the local context. Previous studies have reported that climate emotions such as ‘concern’ are associated with a lack of understanding of the problem (Malka et al., 2009); lack of climate change scientific knowledge (Shi et al., 2016); gender (McCright et al., 2016; Shi et al., 2016); age, country of residence (Stokes et al., 2015); and income (Bronfman et al., 2015). It is assumed that the combination of these demographic variables with factors presented in points (a), (b), and (c), could potentially produce a kind of “planetarian perception mosaic” yet to be studied in a cross-cultural way.

The review of existing relevant literature shows that climate emotional analysis is limited in countries from the global south. Some relevant exceptions are the study by Poma (2018) carried out in Mexico, who identified climate ‘powerlessness,’ ‘fear,’ ‘frustration,’ and ‘guilt’ among people from Mexico DC, interestingly, this author explains that the reasons arousing such emotional states respond to a cultural context characterised by insecurity and economic precariousness. Another study conducted in Ethiopia also emphasises the cultural and environmental context for triggering emotions, where dry seasons expose populations to significant levels of emotional distress, whereas positive emotions such as ‘happiness’ are discussed only with reference to rainy seasons (Cooper et al., 2019). These studies highlight the connection between the cultural and environmental setting and the climatic emotional arousal. Henceforth, further contributions from global south countries would complement current literature in this research field, that is in its own essence global.

Ecuador has more than a decade of experience leading climate change mitigation and adaptation projects in Latin America. Since the establishment of its 2008 constitution, which considers climate change as a state policy, national and international budget has been invested for the creation and implementation of the National Climate Change Strategy (ENCC), as well as for several projects, such as, payments for ecosystem services (*Socio Bosque* Programme), construction of water reservoirs for water governability (*Albarradas*), glaciers retreat adaptation (PRAA), hydro, wind, and solar power generation. Just to mention some examples, these projects succeeded in replacing fossil fuel consumption by 51.78% of renewable energy (ARCONEL, 2021). Socio Bosque achieved the conservation of around 1.58 million hectares of native forests and *páramos*, benefitting more than 120,000 citizens throughout the country (MAE, 2019). These projects, moreover, have received broad media attention generating extensive debate within the country, and have affected public views on a range of climate-related issues (Eisenstadt and West, 2017), and mobilised publics with highly rhetorical (populist) performance and discursivity (de la Torre and Ortiz Lemos, 2016). These particularities make Ecuador an appealing country to analyse the capacity of individuals and collectives to identify their emotions with respect to this climatic issue.

In this context, we aimed (a) to compare the most common emotions aroused toward climate change in European countries and the United States (concern, guilt, anger, scepticism) with an Ecuadorian population sample; and (b) to contribute to the study of less analysed climatic emotions (such as powerlessness, confusion, happiness, optimism, calm). Specifically, we identified the common emotions shared between other geographic contexts and our study area, the demographic variables associated with the emotional arousal, and the reasons underlying such emotional arousal. To do this, we presented to study participants a list of verbal stimuli in the form of a checklist of ten emotional states to select those that best represent their emotional climate reaction. The same verbal stimuli were used to explore basic and complex emotions by analysing the content of the subject’s responses regarding the reasons for their selection. The results are expected to provide deep insights into the role of emotions in public engagement with climate change and how these emotions may vary according to demographic variables.

## Study Area

**FIGURE 1:**
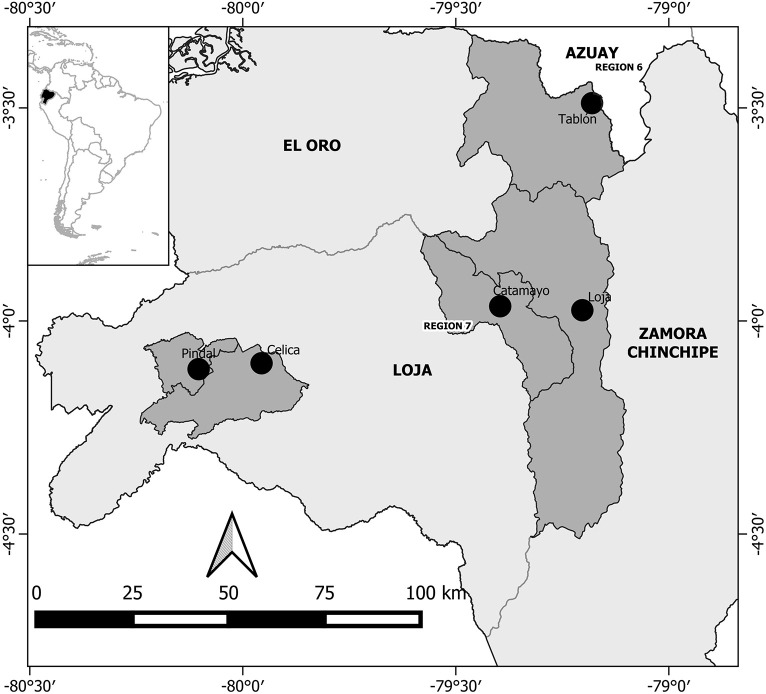
Study areas in dark gray. Black dots show the places where data was collected.

This study focussed on southern Ecuador, and more specifically the province of Loja which has a population of ∼450,000 inhabitants spread in 16 Municipalities (Figure 1). Data were collected from both, urban and rural areas in order to capture potential variation on people’s emotion depending on urban or rural place of residence. The capital city, Loja (∼225.000 inhabitants), whose working population is mainly active in commerce (21%); agriculture (13%), construction (11%), education (11%), and industrial activities (9%) was chosen as urban study area. The study villages of Catamayo (1491 inhabitants), Celica (7947 inhabitants), Tablón (992 inhabitants), and Pindal (6411 inhabitants), were chosen randomly for collecting data from rural areas. These sites are active in agriculture (47%), commerce and services (32%), and construction (7%). The distinction between urban and rural areas in Ecuador is defined according to the presence of basic services, such that ‘urban’ areas have electricity, drinking water, street cleaning, etc.; and ‘rural’ areas do not.

## Method

### Participants

A statistically reliable sample size of 384 people was calculated using the formula for infinite populations at 95% confidence level and confidence interval of 5. For comparative purposes, this sample size was rounded to 400, split into 200 each from urban and rural sites, with 50 respondents for each rural site. Using a simple random strategy, individuals over 18 years old were surveyed in the city during their leisure time in public places such as parks, pubs, churches, bus stations, etc., and in rural areas in parks, after church and market time at weekends and at their homes at different times of the day on weekdays. The sample of 400 subjects was almost balanced between male (57%) and female (43%). The majority of respondents were between 18 and 30 years old (41%) and between 31 and 40 years old (23%). The main occupation profile of rural respondents included professional workers (22%), farmers (21%), and unskilled workers (17%). In urban areas, occupations were mainly distributed among professional workers (34%), and students (27%). Professional workers included scholars/lecturers, schoolteachers, government employees, doctors, nurses, and lawyers. Unskilled workers included masons, business owners, workers, homemakers, and chauffeurs.

### Instruments

A structured questionnaire was designed and piloted with people who shared similar demographic characteristics to the population sample. The questionnaire included a section of demographic questions, a checklist of multiple options of ten emotional stimuli, and an open-ended question to explain the reasons for the stimuli selected. The verbal emotional stimuli (Table 1A) were chosen (a) with the intention to explore the most commonly identified emotions in the existing literature (e.g., concern, guilt) within the cultural and geographical context of our sample, and (b) then with the view of exploring emotions that have been less studied (e.g., anger, confusion, powerlessness, happiness) or have not yet been studied at all in the climatic context (e.g., optimism, indifference, calm). Despite not being an emotion but a cognitive attitude related to belief, ‘scepticism’ was included in the list due to its constant analysis in several studies. The verbal emotional stimuli included basic or “primitive” emotions which are the origin of complex emotions in presence of cognitive evaluation in relation to the self or to others (Johnson-Laird and Oatley’s, 1987; Tables 1B,C). It is worth noting that the verbal label used for the basic emotions described by Johnson-Laird and Oatley’s (1987) correspond to the primary process artificially evoked by activating subcortical brain networks (Panksepp, 1998; Panksepp and Watts, 2011). Basic and complex emotions labels rely on the structure of the language and are not to be confused with the underlying structure of emotions but are to be considered as communicative tools both within the brain and within the social group (Johnson-Laird and Oatley, 1987).

**TABLE 1:** Verbal stimuli used in the questionnaire **(A)**, its type **(B)**, and its description **(C)**.

(A) Verbal emotional stimuli	(B) Type of emotion	(C) Description
Concern	Complex in relation to the self	Anxiety or sympathy for someone else, or something else (Johnson-Laird and Oatley). It implies a timeline and an object of concern. The agent experiencing concern is always the subject.
Guilt	Complex in relation to the self	Shame as result of evaluating one’s past or present performance as morally wrong (Johnson-Laird and Oatley). The guilt person seeks to remedy his/her acts. For this study purposes, guilt refers to actions performed by single or more subjects.
Anger	Basic	Anger is expressed by the subject against something or somebody or somebody’s actions (Johnson-Laird and Oatley). Angry people are highly motivated but also impulsive. Anger could be counterproductive and harmful.
Powerlessness	Complex in relation to the self	Cultural emotion of a self-judged low level of control over a situation. The emotion or feeling of powerlessness implies a negative evaluation of a subject’s power to perform an action; in the case of climate change actions finalized to a solution of the problem, sometimes the subject declares the reasons or the agents at the root of her/his feeling (TenHouten, 2016).
Confusion	Complex in relation to the self	It is a cognitive disequilibrium caused by contradictions, conflicts, erroneous information, can be beneficial to learning if appropriately induced, regulated, and resolved (D’Mello et al., 2014).
Optimism	Complex in relation to the self	Happiness from positive evaluation of events in relation to one’s goals (Johnson-Laird and Oatley).
Happiness	Basic	Pleasant emotion of satisfaction about fulfilling desires, therefore is conditional and a temporal relaxation state of ‘hope,’ ‘joy,’ ‘calm,’ ‘optimism,’ delight, etc., for achieving something
Calm	Generic	Not in extreme state of emotion (Johnson-Laird and Oatley). Calmed people accept things as they are.
Indifference	Complex, in relation to others	Not caring for (Johnson-Laird and Oatley).

The open-ended question was added because most of the studies in this research field use close-ended questions to select a type of emotion or a phrase/message that refers to an emotion felt without any reference to its causative agents. Yet, an in-depth understanding of the reasons arousing emotions is mandatory for organising climate campaigns and activities (Moser, 2010). Henceforth, participants had the agency for explaining by themselves in a short-written sentence the motivations/reasons or causative agents of their choice.

Structured questionnaires were administered between 04/2014-01/2015. Subjects were asked to complete the questionnaire individually. Initially, they were asked to answer demographic questions. Subsequently, they were asked to read and reflect on the checklist of the ten verbal emotional stimuli, and to select one or multiple stimuli that best reflect their climate emotions. Eventually, the following open-ended question was repeated in the same way to all subjects: “would you please explain why you feel the emotion you have selected?”

### Data Analysis

The data collected through the checklist were analysed by using the statistical package IBM SPSS 22, with Chi-square tests conducted to identify associations between the emotions selected and demographic variables. Responses provided to the open-ended question were manually coded and organised for pattern detection and subsequent content analysis, this included counting and categorising elements according to the type and number of times a particular set of codes (words) were repeated (after Saldaña, 2018).

Subjects provided between one and four causal reasons when answering the open-ended question. In order to avoid inflating the percentage results corresponding to multiple causal reasons provided by a single subject, we considered only the first causal reason expressed, arguably the most important one for the subject. The analysis proceeded as follows: (1) Researcher DLB analysed a subset of answers chosen randomly from the data universe (30 for each emotion), she was blinded about respondent’s age, occupation, sex, and place of residence (rural or urban). The aim was to identify a set of mutually exclusive categories based on a single concept (Weber, 1990), thus any response had to be assigned to only one category, e.g., the category “health” includes any reference to health problems such as ‘diseases’ or ‘generic health damage’ (Table 2). Such responses were not included in any other category but to ‘health.’ Afterward, the categories were discussed and shared among two of the authors (VI-G and DL) until 100% agreement was reached. Finally, both authors assigned each response to a specific category independently from each other and blindly about the subjects’ demographics. Later, authors compared their results and checked if any single response had been assigned to the same category, in this case the response was accepted as a valid datum; responses assigned to different categories by the authors were discarded, without any further discussion and/or negotiation. In Table 2 are presented some examples that correspond only to the results that were agreed upon by both researchers.

**TABLE 2:** Examples of the causative agents provided by study participants for experiencing the six most selected emotions.

Emotional states	Categories	Examples of participants (translated from Spanish by the authors)	% of responses
Concern *n* = 295 responses	Descendants and future generations	“*We don’t know what life our children, grandchildren, etc. will have*” “*For the children to come*”	14
	Weather changes	“*Climate is changing everyday*” “*One can notice that climate is changing*” “*high temperatures worldwide or the opposite, a lot of rainfall*”	14
	Unfriendly behaviour	“*There are people who are not environmentally conscious*” “*People find it hard to change” “If people don’t change their life pattern, the future will be more difficult” “People have no intention to stop pollution*”	14
	Health	“*Skin cancer*” “*it’s affecting people’s health dramatically*” “*There are more diseases*” “*It will affect our life quality and health*” “*It will affect our health*”	13
	Human survival	“*It affects us directly, both in terms of health and survival*” “*Every time the climatic changes are stronger and with greater damage to human life and to nature itself*” “*The future of humanity is at stake*”	9
	Planet destruction	“*The planet is tearing apart*” “*because of all life forms existing in our planet*” “*I live here and there’s no other planet to live in*” “*There is much danger that our planet will be destroyed*”	9
	Negative outcomes	“*It could be unbearable*” “*The coming generations will not necessarily succeed in reversing the consequences*” “*I think the effects will be very serious*” “*one day everything will be over because of climate change*”	9
	Uncertainty	“*We don’t know what it’ll happen*” “*we do not know what kind of disasters will come and that leads us to an uneasines*s” “*no one knows what it’ll come next*”	8
	Natural disasters and water shortage	“*In time there will be water shortages*” “*Because of the natural disasters we are currently going through*” “the water and air is going to run out”	7
	Generic effects	“*It could affect rural development*” “*It worries me that it could affect my future*” “*Every day is affecting us more*”	4
	Agriculture and food shortage	“*It affects our food*” “*There’ll be food shortage*”	6
	Governments, industrialised countries	“*Industrialised countries make excess CO2*” “*No action is taken to reduce pollution; especially by governments*”	2
Guilt *n* = 136 responses	My fault (first person of singular)	*“I use aerosol cans” “I generate waste” “I use vehicles and electronic devices that pollute” “I travel by plane” “I do not recycle” “Because of my lifestyle” “I am consumerist” “It is my fault, my responsibility”” I deforest” “I use agrochemicals”*	34
	Our fault (first person of plural)	*“We use chemicals for our crops” “we are responsible for what is happening” “we are the big polluters”*	32
	I don’t help	*“I say nothing to the authorities” “I feel so limited about doing anything” “I don’t educate people” “I don’t lead environmental campaigns”*	13
	Third person (singular)	*“One contributes to global warming” “one causes damage”*	9
	Don’t do enough	*“I could do much more, but I don’t” “We could do more to make it better” “I know I could do more”*	6
	Third person (plural)	*“Human beings are guilty” “Everybody’s fault” “humanity is polluting” “Man is guilty”*	2
	Can’t help it	“*I can’t cooperate at improving the environment’s health*”	1
	Don’t do anything	“*We do anything about it*” “*we do nothing to make it better*”	1
Powerlessness *n* = 93 responses	I can’t do much	“*Because I can’t help much*” “*Recycling is not enough*” “*one can’t help much with climate change*” “*Within my means I cannot do more than I would like to*”	30
	I can’t change it	“*Not being able to change with all that contributes to climate change*” “*Campaigns and rules do not change anything*” “*Can’t make a difference*” “*my change doesn’t make others change*”	14
	Lack of awareness	“*People don’t realise that they are the main culprits for doing nothing*” “*The actions taken are very few and there is no real awareness of the damage being done*” “*There is not social awareness*” “*People don’t understand*”	14
	I can’t do anything	“*I want to, but I can’t do anything*” “*one can do nothing to repair these damages*” “*There is no major corrective mechanism that I can implement*”	12
	I can’t oblige others	“*You can’t do anything if they all pollute*” “*It is a giant task to educate humanity to take care of the planet*” “*I can’t force people to take care of the environment*”	8
	I can’t stop industries	“*We cannot stop the great world powers of industry*” “*Part of the blame lies with the big companies that only think about capital no matter how much damage they cause*” “*Because of the power that the great industrial powers have and do nothing to change this evil*”	6
	Don’t know what to do	“*One doesn’t know what can be done to change those big, global problems*” “*I don’t know what to do with the population growth, more food is needed, and more forests are destroyed*” “*not knowing what to do so that we all take care of the environment*”	5
	I don’t’ have the power	“*I do not have the power to regulate laws*” “*I can’t reach high levels of society*” “*I do not have the decision to prevent further contamination of the environment*” “*Nothing can be done about the attitude of upper-class people, businessmen, powerful countries that are at war and producing atomic bombs*”	3
	Government’s fault	“*Governments are not doing what they should*” “*governments and politicians do not take drastic measures to protect the environment*”	2
	Nature’s reaction, fear	“*One day, nature will get tired of so much pollution and will act*” “*one does not know what is happening or what might come in the future*”	2
	I do nothing	“*We do nothing to make the government listen to us and change the climate*”	1
Anger *n* = 89 responses	People (humankind)	“*There are people who can pollute less but they don’t, they misbehave*” “*I keep seeing people who pollute the environment*” “*People know and don’t want to change about it*”	46
	Governments and Politicians	“*Governments do not take measures to remedy or stop the contamination*” “*Politicians who should be doing much of the work do nothing or almost nothing about it*” “*The municipality, the President, does not help with the problems with our crops*”	18
	Ourselves	“*I think it’s not enough what we do to stop climate change*” “*We do not do what is humanly possible to solve the problem*” “*instead of stopping pollution, we keep doing it*”	8
	My self	“*I am not part of that movement of consciousness of what is happening day after day*” “*I don’t have the vote to make efficient decisions*” “*I help depleting resources*”	6
	Industrialised countries	“*The great powers are to blame*” “*developed countries that do nothing to avoid polluting the planet*”	6
	Health effects	“*It affects my health and my activities*” “*I’m ill*” “*it affects those of us with unstable health*” “*those of us who are older are affected because of our health*”	5
	Weather changes	“*The suns are very strong and when it rains, it rains very hard*” “*the changing weather makes me sick*” “*when you go out to have fun and then the day changes*”	5
	Industries and big corporations	“*Instead of worrying about the ecology, there are foreign companies exploiting resources*” “*No one does anything to defend nature against mining and logging*” “*big industries destroy much more*”	4
	Potential effects	“*The costs for agriculture are high, they sell us expensive things*” “*Because of the serious consequences and disasters*”	2
	Generic answers	“*There’s no solution*” “*You can’t go out and do your activities*” “*there’s no going back*” “*some people are not to blame and suffer the consequences*”	7
Confusion *n* = 39 responses	Lack of knowledge	*“There is a disagreement between anthropogenic and natural causes” “I don’t understand much about this theme” “I don’t know how my actions will affect the climate” “I don’t know the reasons why people don’t decide to change their mind” “I don’t know how to influence others to make them aware”*	54
	Weather changes	*“Weather is changing” “People are confused about crop blooming” “I don’t really understand what’s going on with the weather” “I don’t know when it will rain or when it’ll be sunny”*	28
	Uncertainty	*“We should not fall in the trap of green capitalism” “I don’t know when the warming will end” “We don’t know what will happen”*	10
	Debate and political decision	*“There are many positions and the decisions made are not effective to address climate change” “I don’t understand the political decisions taken by the Ministry and other environmental organisations”*	8
Optimism *n* = 34	New behaviour	*“I am hopeful that people will change their behaviour” “Although it has not been enough, there has been a change in people’s mentality about the problem”*	44
	We can make it	*“Campaigns can make it better” “Every cloud has a silver lining” “I know that this has to stop” “we are still on time to contribute with two cents and fight against this phenomenon” “We don’t have to sit back and do something”*	26
	New knowledge and technology	*“There are scientists in the world creating technology” “There is more and more knowledge” “These forces us to improve and innovate environmentally friendly technologies.*	12
	New generations	*“New generations are more aware and know ways to avoid climate change” “With the new education provided we raise awareness in children so that they are aware and can do something about it”*	9
	New politicians	*“There are politicians who can make the change”*	6
	Various	*“I know that with God’s help this may change”*	3

## Results

### Participant’s Most Selected Emotions Toward Climate Change

**FIGURE 2:**
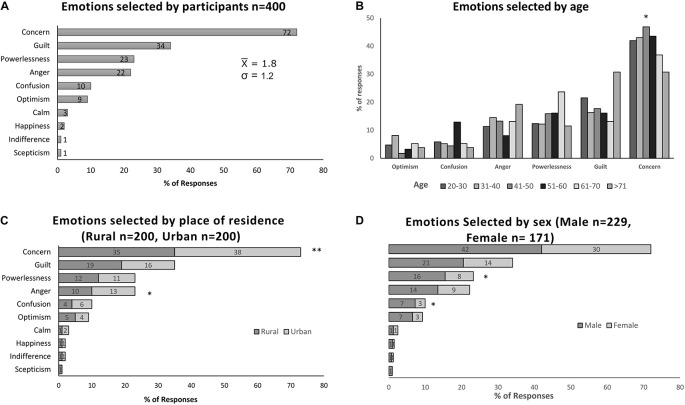
**(A)** Participant’s most selected emotions, **(B)** emotions associated with age, **(C)** emotions associated with place of residence, **(D)** emotions associated with sex. *p < 0.05; **p < 0.005.

Most of the participants (*N* = 400) selected ‘concern,’ followed by ‘guilt,’ ‘powerlessness,’ ‘anger,’ and ‘confusion.’ The least selected emotions were ‘indifference’ and ‘scepticism’ (Figure 2A). Participants selected mostly just one emotion (52%) or maximum two emotions (23%) from the list. The average number of emotions selected for the entire sample was 1.8 with a standard deviation of 1.2.

The Chi-square test conducted for detecting associations between participant’s demographic background and the emotions selected from the list, produced significant values between age, place of residence and the emotion of ‘concern.’ Specifically, the analysis indicate that more subjects between 41 and 60 years old [χ^2^(4,400) = 15,239, *p* < 0.004], and from the urban sector [χ^2^(1,400) = 7,741, *p* < 0.004] selected this emotion (Figures 2B,C). Likewise, the test produced significant values between sex and ‘powerlessness’ with more men selecting this emotion [χ^2^(1,400) = 4,390, *p* < 0.023] (Figure 2D). Another significant association was found between the place of residence and ‘anger’ where more subjects from the urban sector selected ‘anger’ [χ^2^(1,400) = 3,260, *p* < 0.046] (Figure 2C). Finally, the test found a significant association between ‘confusion’ and sex indicating that disproportionately more male respondents [χ^2^(1,400) = 4,223, *p* < 0.028] selected this emotion (Figure 2D).

### Origin/Cause of the Emotions Selected

The responses given to the open-ended question produced a wide variety of causative agents for the emotions analysed (Table 2). Participants who selected ‘concern’ provided 295 reasons that were grouped into 12 categories. The main reasons for this emotional state refer to a concern for the *future generations* (14%), weather changes (14%), *people’s unfriendly behaviour with the environment* (14%), and *health* (13%). It is worth noting that, participants who identified weather changes as a reason of concern were predominantly rural residents, whereas those concerned with people’s unfriendly environmental behaviour were mostly urban residents. Participants who selected the emotional stimulus ‘guilt’ provided 136 different reasons for this, which were subsequently grouped into eight categories. Thus, most of the respondents position themselves as the responsible actor for climate change, either individually (34%) or collectively (32%). Others referred to limited actions undertaken by humans (thus using the third person plural), to tackle climate change as the main reason of the emotional state of guilt. It is important to mention that although no significant difference was found in the Chi-square analysis between selecting ‘guilt’ and the demographic variables, the responses to the open-ended question reveal differences in the reasoning of guilt. Thus, farmers explained that they feel ‘guilty’ because they *deforest or use agrochemicals for their crops*, whereas urban respondents felt ‘guilty’ because they *pollute the environment and have a consumerist lifestyle*.

People who selected ‘powerlessness’ provided 93 causative agents for their response subsequently grouped into 10 categories. The most common reason leading to this emotional arousal was a sense of helplessness for *“not being able to do much about climate change”* (30%), or *“change it”* (14%), or simply because *“there is little social awareness”* about this phenomenon and its potential effects. Participants who selected anger, provided 89 causative agents for their response which were grouped into 17 categories. The most common reasons for feeling angry were *“people’s lack of awareness,” “selfishness”* and *“behaviour”* (46%), *“governments and politicians”* (18%). Other types of reasons used the first person plural (we) or singular (I), to refer their anger toward humans (8%) or themselves (6%).

People who selected ‘confusion’ provided 39 causative agents that were grouped into eight categories. The main reasons causing such emotional arousal were the lack of knowledge (51%) and perceived weather changes (26%). The lack of knowledge involved responses about the causes and effects of the phenomenon, but also provided interesting responses indicating *“lack of knowledge to influence others to change their thinking,”* or about the ways in which they can *“help individually.”* Regarding ‘weather changes’ these were mainly mentioned by farmers, who indicate that they feel confused because they cannot longer *recognise “dry and wet seasons,”* or because they do not follow *“crop blooming.”* It is important to mention that the Chi-square test did not find a significant association between ‘confusion’ and occupation but suggests an influence of the environment on the emotional arousal.

People who selected ‘optimism’ provided 34 causative agents that were grouped into six categories. Most reasons indicate that they are optimistic because they feel that people are adopting or will adopt more climate-friendly behaviour (41%). They also mentioned that people are *“reforesting,” “taking more responsibility,”* or simply *“they are hopeful that people will change their behaviour.”* Other reasons used the first person plural to denote that *“we can make it”* (26%) and must *“do our bit,”* while others mention that there are *“campaigns* and *options that will make it better.”* Other participants were optimistic that *“new knowledge and technology”* will help to deal with this problem (12%), while others were confident that *“new generations”* will make a change (9%). Some were even confident that *“new politicians”* (6%) or *“god”* will make the difference (3%).

The responses provided for the least selected emotions, namely ‘calm,’ ‘happiness’ ‘indifference’ and ‘scepticism’ (which together add up to 7% of the emotions selected) denoted a low arousal reaction in that people who selected these emotions believe that *“everything will be alright”* or that they will *“get used to new climatic conditions,”* or because they do not think global warming will affect them much (Table 3). As for scepticism, it is worth noting that, apart from being the least selected emotion, the self-reported reasons for feeling sceptical are not related to the debate on the existence of the phenomenon, but rather denote apathy to what is happening or display mistrust that “*major global powers will do anything about it”*.

**TABLE 3:** Examples of the causative agents provided by study participants for experiencing the least selected emotions.

Emotional states	Examples of respondent’s answers (Translated from Spanish by author)
Calm *n* = 10	*“I still breath fresh air” “There’s nothing to change” “It won’t affect me that much” “We will used to new climate and changes” “When times like this come, we must remain calm” “We will get used to weather changes” “As long as we have God nothing will happen” “climate has always changed” “Trees are being planted” “Kids are being educated”*
Happiness *n* = 5	*“There are many consequences about climate change that we cannot be certain about” “Humanity is becoming aware to stop global warming” “There’s nothing we can do against god’s will” “I’m with my kinds, that’s enough”*
Indifference *n* = 3	*“Everything will be alright” “There’s not much we can do about this problem” “I don’t think it will affect me that much”*
Scepticism *n* = 3	*“The major world powers will do nothing” “No other forms of energy beyond solar, wind, etc. can be generated.” “Global warming has already happened, although there are reasonable explanations for this, the science must always be critically analysed”*

## Discussion

Addressing climate change demands sacrifices or deep changes in our lifestyles (Hulme, 2013), toward which the public may be unwilling to adopt measures with immediate economic consequences, as happened with farmer’s protests in France in November 2018 following the announcements of diesel price increases, or, on the contrary, may demand from government major interventions as climate activist groups do. Such behaviour requires an emotional analysis in that emotions are advocated as key factors in the mobilisation of energies necessary for this task (Moser, 2010). However, emotions depend on many variables such as the media, political orientation, and demographics, that differ between nations and social groups. This forms a mosaic of multiple pieces that requires studies across different social, cultural, and political contexts to obtain a more comprehensive understanding of the climate emotional arousal. In this sense, this study provides data collected in Ecuador, a country that might be a key example in that it has implemented a variety of climate strategies impacting societies at the individual level (e.g., preferential electricity rate on households whose stoves have been switched from gas to induction) and the collective level (e.g., payments for forest conservation).

### Analysis of the Climate Emotional Arousal

‘Concern,’ ‘guilt,’ ‘powerlessness,’ ‘anger,’ and ‘confusion’ were the participant’s five most selected emotions. Interestingly, two of these emotions (powerlessness and confusion) have been scarcely analysed in studies conducted in Europe and the United States, providing further evidence on the importance of local context and demographics on exploring emotional arousal to climate change.

The scarce evidence on the relationship between ‘powerlessness’ and climate change come from two very different geographic, political and economic contexts, namely New Zealand (TenHouten, 2016) and Mexico (Poma, 2018), suggesting an interesting international trend that deserves to be studied further in other countries. More interestingly, the reasons for experiencing ‘powerlessness’ are similar between our study participants, who experience climate change mainly as an *out-of-control* situation over which they cannot do much or cannot change, and those reported by New Zealanders, who feel that climate change is too big for their actions to have an impact or to change the outcomes, and by Mexicans, who experience powerless because they believe climate change has no solution or because people on upper circles have a greater impact in generating changes to address. Our results found more male respondents. Despite these interesting similarities, both studies did not explore the potential influence of demographics on such emotional arousal. In contrast, our study indicated that males selected more often ‘powerlessness’. ‘Powerlessness’ is provoked by a self-judged low level of control over a situation (Ajzen, 1991), and although it has been identified as secondary to other factors in explaining climate action (Aitken et al., 2011), people who feel more powerless are judged less likely to act and to consider climate change as an important factor when changing behaviour (Aitken et al., 2011). Therefore, it is of vital importance for a climate campaign to avoid arousing ‘powerlessness’ in its messages as well as to consider the demographics involved.

Regarding ‘confusion’ the evidence sustains that this emotional state is common when exploring climate change risk, knowledge, and perception, and it is associated with lower levels of climate action (Aitken et al., 2011; Barnes et al., 2013). Most of the studies analysing ‘confusion’ focus on people’s climate change scientific knowledge and on the sources producing misconceptions and contradictions (McCaffrey and Buhr, 2008; Plutzer et al., 2016; Selseng et al., 2021). Less clear is whether people are confused for other reasons beyond their knowledge on the topic. Our study found that in addition to knowledge gaps, people feel confused because they *“do not understand why people do not change their mind and take actions,”* or because of *“changing weather patterns.”* Furthermore, ‘confusion’ affects men and women differently with more males feeling confused.

‘Concern’ was the participant’s most selected emotion, similar to studies in other countries (e.g., Brulle et al., 2012; Smith and Leiserowitz, 2014; Hardesty, 2015; Leiserowitz et al., 2020). However, we found some differences regarding the factors associated with climate concern. Our results indicate that people between 41 and 60 years old and from urban areas tend to experience more concern about climate change. These differ from previous studies which indicate that young people tend to be more concerned about climate change (Stokes et al., 2015). Furthermore, our study did not reveal any associations between ‘concern’ and gender, unlike McCright (2010); Shi et al. (2016), and Stevenson and Peterson (2016) who found women to express more ‘concern’ about climate change. This may be explained by the claims by Eisenstadt and West (2017) who assert that Ecuadorians have developed a series of climate concerns thanks to governmental campaigns and media coverage designed to reach all audiences.

Moreover, our data obtained from the open-ended question provided empirical evidence of the origin or cause of the emotional state of concern. The results found ‘concern’ to be largely experienced in the context of *“future generations,” “weather changes,” “people’s environmental unfriendly behaviour”* and *“health.”* It is noteworthy that while in other countries, public concern for future generations was very common in the 1980s (Capstick et al., 2015), in the case of Ecuador it is still strongly present. As for the public’s concern about weather changes, a study by Brulle et al. (2012) indicates that weather events, in themselves, do not influence levels of public climate concern, nonetheless our results indicate that ‘weather changes’ are the second main reason leading to experience this emotional response.

Overall, our findings show that climate public concern in southern Ecuador is strongly based on future scenarios that include primarily a sense of legacy, place disruption, other people’s behaviour, and the very health of the participants. These types of responses are encouraging if we consider that it is critical to engage people in envisioning a future worth fighting for (Moser, 2010). Adding the fact that ‘concern’ encourages climate action (Stevenson and Peterson, 2016), as well as responsible environmental behaviour (Bronfman et al., 2015), or support for global warming policies (Smith and Leiserowitz, 2014), climate campaigns appealing for concern may well accompany their messages with the feasibility of individual actions and their impact on generating collective change.

‘Guilt’ was the second most selected emotion by study participants. Our data revealed a strong sense of regret about their *lifestyle* but also about the *little or no action taken*. In fact, certain responses mentioned feeling guilty for *not complaining to the authorities*, a deeply collective and activist thinking that has not been mentioned in other countries. Although the Chi-square analysis did not provide significant associations between selecting ‘guilt’ and the demographic variables, the data from the open-ended question indicate that while farmers feel ‘guilty’ because of the *“agrochemicals they use,”* urban respondents do it because they *“pollute and have a consumerist lifestyle.”* This highlights the role of the local environment and the relevance of having more local data that helps climate campaigns build varied messages to reach different audiences, even within the same region and nation. Previous studies looking at the origin of climate guilt, indicated some similarities with our Ecuadorian sample. A study from Mexico mentions that with more information people feel guilty about the things they buy (Poma, 2018), while in Norway people tend to feel guilty about their privileged lifestyles (Noorgard, 2011). Self-reported consumer practices are indeed regarded as climate-damaging behaviour particularly for the Global North (Neckel and Hasenfratz, 2021). In the United States guilt arises with the subject’s concrete experiences and when climate change is perceived close to s/he (Chu and Yang, 2019). Doherty and Clayton (2011), claim that ‘guilt’ is associated with climate change in that the process of grief and mourning help individuals to make more ecologically stable life choices, whereas Garvey (2010), appeals to feelings of guilt to motivate climate action. Indeed, ‘guilt’ is considered as a constructive reaction enhancing pro-social behaviour to amend damage (Baumeister et al., 1994; Niedenthal et al., 1994). However, people make amends for what they feel guilty for only when they believe in the efficacy of their actions (Brügger et al., 2015). It is worth mentioning that guilt has a variant emotional state called “shame,” which was outside the focus our study. A further research analysing this issue could be relevant in that ‘shame’ could be destructive and stop actions.

‘Anger,’ one of the two basic emotions analysed, was the fourth most selected by study participants. Our results revealed that people feel angry mainly *with other people*, *governments, politicians*, and with *themselves*. The arousal is caused by a societal behaviour that favours pollution, politician’s passive attitudes, and individual failure to change or slow down climate change. Climate anger has also been identified as a common emotional state in previous studies (e.g., Myers et al., 2012; Smith and Leiserowitz, 2014; Chu and Yang, 2019; Neckel and Hasenfratz, 2021). However only few of them identify the potential causative agents for such emotional arousal. Chu and Yang (2019) found in the United States that anger arises when people recognises that climate change is no longer distant and uncertain threat, whereas Myers et al. (2012) found in the same country that, ‘anger’ is aroused among people at the two extremes of the “global warming six Americas” scale, that is, among those who are “alarmed” and convinced of the reality of climate change and those who are “dismissive.” Clearly, our Ecuadorian sample is not dismissive -only a marginal 1% of the respondents selected ‘scepticism’- Furthermore, our data obtained in the open-ended question indicated that ‘anger’ is aroused by a sense of frustration at the *“society’s passivity”* and the *“impassiveness of its government representatives to take actions.”* Our results, furthermore, indicate that more urban residents feel angrier. We believe that this particular deserves some attention in the design of climate campaigns. ‘Anger’ is an emotion that energises the person and motivates action. Some researchers even claim ‘anger’ to have a positive influence on the intention of making sustainable consumption choices (Wang and Wu, 2016). According to Johnson-Laird and Oatley (1987) angry people are highly motivated, but also impulsive, this might explain previous claims arguing that people feeling ‘anger’ often make poor choices that can be self-defeating (Baumeister and Bushman, 2011), leading individuals to a less prosocial behaviour (Drouvelis and Grosskopf, 2016).

Our results found that positive emotions such as ‘optimism’ ‘happiness’ and ‘calm’ are aroused by sentiments of hope that people will change their attitudes and become more climate responsible, just as they are confident that future technology and knowledge will help to deal with climate change cause and impacts. Positive emotions such as happiness, relaxation and optimism are indeed regarded to prepare individuals for hard times, helping to develop flexibility, creativity, and problem-solving ability (Sutton and Douglas, 2013). ‘Hope’ emerges as a much more significant predictor of pro-environmental behaviour (Stevenson and Peterson, 2016), in particular form of ‘constructive’ hope that is linked to a high degree of self- perceived efficacy (Ojala, 2015).

Finally, it is important to note that in our study ‘scepticism’ or ‘indifference’ were hardly selected, in contrast to studies in other countries such the United States, where 12% of the population is doubtful and 8% is dismissive (Leiserowitz et al., 2020). Capstick et al. (2015) observed that in some parts of the world there was a growth in public scepticism about climate change since the late 2000s attributed to a range of factors such as the global financial crisis. This trend could be repeated with the arrival of Covid-19.

Succinctly, ‘concern’ ‘hope’ and ‘anger’ are associated with higher levels of action (Myers et al., 2012; Ojala, 2012, 2015; Stevenson and Peterson, 2016), whereas ‘shame,’ ‘guilt,’ ‘powerlessness,’ and ‘confusion,’ are associated with lower levels of action (Heyd, 2010; Aitken et al., 2011; Barnes et al., 2013; Brügger et al., 2015; Caillaud et al., 2016). In considering that our study participants experience more than one emotion toward climate change, and that the most selected were ‘concern,’ ‘guilt,’ and ‘powerlessness,’ it is important to understand that the construction of climate narratives must look at the set of emotions experienced before appealing to a single specific emotion. That is, while concern is seen as an emotion that can lead to action, combining it with feelings of powerlessness can have the opposite result. Moreover, the geographic and demographic context should also be considered when climate action messages are intended to be broadcasted, in that behavioural intentions based on emotions are culturally based (Caillaud et al., 2019). Emotions have a collective influence in that a person can experience an emotion without direct or personal involvement, requiring only that the individual self-recognise as a member of a group (Caillaud et al., 2016).

## Conclusion

The present study obtained an in-depth insight of the causal reasons/motivations or agents that trigger any particular emotion. By means of an open-ended question, we stimulated participants to disclose their thoughts and feelings to furnish a more complete scenario on the mostly felt sensitive aspects aroused by climate change. Our research indicates that Ecuadorians share feelings of ‘concern’ and ‘guilt’ with other nations, and differ from them in that they feel powerless, angry, and confused, but not at all sceptical. The results also found that the causative agents for the emotional arousal differ from that found in other countries and are associated with demographic variables and influenced by the cultural environment.

Although we identified the most selected emotions and their causative agents, we have some limitations to reveal participant’s real action tendencies, as this was not part of the scope of our study. We conclude that such a complex phenomenon as climate change, whose appraisal is influenced by the media and by the public debate, elicits a panel of emotional reactions, and an even wider panel of perceived priorities. In fact, all the verbal stimuli proposed arouse answers that divide any group of respondents into smaller subgroups who prioritise different aspects or problems and give us a much wider and richer view of the individual and social reactions to climate change. This should be the starting point in the development of climate campaigns that should focus and include a complete panel of perceived problems in relation to climate change in order to avoid stereotypes and enhance effectiveness. In our opinion, the focus of previous studies exploring specific emotions such as ‘concern’ or ‘guilt,’ have been reductionist in defining how a person engages emotionally with climate change, and that this may interfere in the development of more robust climate campaigns. We stress that research on climate emotions needs to allow participants to express the reasons underlying the emotional arousal. We also conclude that in taking this approach, the influence of age, the place of residence, and gender, appears to differ from those found in other geographical contexts, which further indicates that climate campaigns and policies should be developed by considering regional demographics, as we highlight the importance of conducting cross-cultural and *trans*-regional parallel studies.

## Data Availability Statement

The datasets presented in this article are not readily available because the raw data supporting the conclusions of this article will be made available by the authors only for future joint work between authors and stakeholders. Requests to access the datasets should be directed to VI-G, mviniguez1@utpl.edu.ec.

## Ethics Statement

Ethical review and approval was not required for the study on human participants in accordance with the local legislation and institutional requirements. Written informed consent for participation was not required for this study in accordance with the national legislation and the institutional requirements.

## Author Contributions

VI-G worked on writing, methodology, data collection, analysis, discussion, and reviewing the manuscript. DL collaborated on writing, methodology, analysis, discussion, and reviewing the manuscript. JT collaborated reviewing the manuscript as well as checking for spelling. All the authors contributed to the article and approved the submitted version.

## Conflict of Interest

The authors declare that the research was conducted in the absence of any commercial or financial relationships that could be construed as a potential conflict of interest.

## Publisher’s Note

All claims expressed in this article are solely those of the authors and do not necessarily represent those of their affiliated organizations, or those of the publisher, the editors and the reviewers. Any product that may be evaluated in this article, or claim that may be made by its manufacturer, is not guaranteed or endorsed by the publisher.
